# *Coxiella burnetii* Endocarditis and Meningitis, California, USA, 2017 

**DOI:** 10.3201/eid2408.180249

**Published:** 2018-08

**Authors:** Lao-Tzu Allan-Blitz, Ashyln Sakona, William D. Wallace, Jeffrey D. Klausner

**Affiliations:** David Geffen School of Medicine at the University of California Los Angeles, Los Angeles, California, USA (L.-T. Allan-Blitz, A. Sakona, W.D. Wallace, J.D. Klausner);; Fielding School of Public Health at the University of California Los Angeles, Los Angeles (J.D. Klausner)

**Keywords:** Coxiella burnetii, bacteria, Q fever, endocarditis, meningitis, meningitis/encephalitis, epidemiology, human infection, California, United States

## Abstract

The epidemiology of *Coxiella burnetii* infection in the United States is not well characterized. We report a case-patient with *C. burnetii* endocarditis and meningitis. Infection was diagnosed by detecting high serologic titers for *C. burnetii* and confirmed by sequencing of *C. burnetii* 16S rRNA isolated from resected valvular tissue and PCR of cerebrospinal fluid.

*Coxiella burnetii* is the bacterium responsible for Q fever. The epidemiology of *C. burnetii* infection in the United States is not well characterized. Chronic infection can result in endocarditis or other complications. We report a case-patient with *C. burnetii* endocarditis and meningitis.

## The Case-Patient

A 38-year-old man with no unusual medical history was hospitalized in 2017 for evaluation of a new cardiac murmur detected by his primary care physician. The man lived near the Pacific coast of central California, USA, where he worked from home as a broker selling produce from local farms. He had not visited those farms, but cattle were raised on properties neighboring his home. Three months before hospitalization, he had an episode of bronchitis treated with azithromycin; his symptoms resolved. A few weeks before that illness, he visited a petting zoo with his children where he was exposed to goats and chickens. The patient was not aware of any parturient animals at the zoo. Other animal exposures were limited to his 2 pet dogs. He denied ingestion of unpasteurized dairy products, a history of injection drug use, or recent travel.

Six weeks before hospitalization, a tender nodule developed on the palmar aspect of his left fifth digit. That nodule resolved without intervention, but severe right midfoot pain and swelling developed. The pain and swelling were diagnosed as cellulitis or gout, and the patient was given trimethoprim/sulfamethoxazole and indomethacin.

Two weeks before admission, fever and severe headache developed, and the man visited the emergency department of another hospital, where a lumbar puncture was performed. Cerebrospinal fluid leukocyte count was 253 cells/μL with 52% lymphocytes and 43% neutrophils, glucose level was 35mg/dL, and protein level was 63 mg/dL. Results for a FilmArray Meningitis/Encephalitis Panel (BioFire Diagnostics, LLC, Salt Lake City, UT, USA) were negative. He was discharged and given a diagnosis of aseptic meningitis believed to be secondary to treatment with indomethacin.

On follow-up with his primary care doctor, the patient reported ongoing fevers, chills, and drenching night sweats for 2 weeks and a 10-pound weight loss in the preceding 2 months. He was admitted to another hospital, where a new cardiac murmur was detected. A transthoracic echocardiogram showed vegetations on the mitral valve. He was then transferred to Ronald Reagan University Medical Center (Los Angeles, CA, USA) for a higher level of care and surgical evaluation.

At admission, he was afebrile and had unremarkable vital signs. A physical examination showed a harsh holosystolic murmur, a decrescendo diastolic murmur, and a splinter hemorrhage. A transesophageal echocardiogram showed a bicuspid aortic valve with thickened, calcific leaflets and severe regurgitation and a mobile vegetation attached to the mitral valve cordae with subvalvular calcifications.

Admission blood cultures and cultures obtained at the previous emergency department visit were negative for bacteria. He was given empiric vancomycin and ceftriaxone and underwent an aortic valve and aortic root replacement and mitral valve repair. At the time of surgery, multiple sets of blood cultures remained negative for bacteria. Intraoperatively, chronic changes in the aortic root near the right coronary cusp were observed and believed to be suggestive of a previous endocarditic process with a healed area of disruption. Calcific lesions involving the mitral subvalvular apparatus were resected and tested by pathologic analysis.

On postoperative day 1, serologic analysis for *C. burnetii* showed complement fixation titers (IgG phase 1, 1:2,077,152; IgG phase 2, 1:8,388,608; IgM phase 1, 1:1,024; and IgM phase 2, 1:2,048). Pathologic evaluation of the valvular specimen showed multiple fragments of tan/red to tan/brown soft tissue with focal hemorrhage and calcifications. Microscopic evaluation showed valve tissue mononuclear cells. Gram staining showed numerous small clusters of gram-negative coccobacilli, consistent with an intracellular distribution (Figure).

**Figure:**
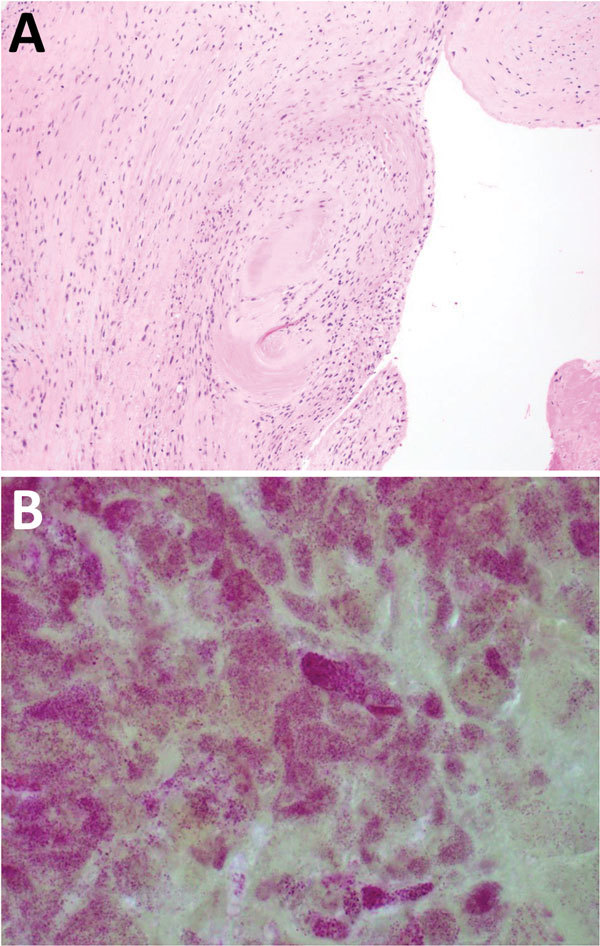
Results of testing for a 38-year-old man with *Coxiella burnetii* endocarditis and meningitis, California, USA, 2017. A) Cardiac valve tissue showing fibrous scar and chronic inflammation (hematoxylin and eosin stain, original magnification ×100). B) Numerous clusters of gram-negative cocci are consistent with intracellular organisms (Gram stain, original magnification ×1,000).

Subsequent 16S rRNA gene sequencing confirmed the presence of *C. burnetii.* Retrospectively, a qualitative PCR on stored cerebrospinal fluid and resected valvular tissue showed a positive result for the 127-bp insertion sequence 1111, consistent with *C. burnetii*. The patient was given doxycycline and hydroxychloroquine for a planned course of 18 months.

He is still receiving therapy and serial complement fixation titers have decreased. After 5 weeks of therapy, his titers were IgG phase 1, 1:262,144; IgG phase 2, 1:1,048,576; IgM phase 1, 1:512; and IgM phase 2, 1:1,024. After 10 weeks of therapy his titers were IgG phase 1, 1:32,768; IgG phase 2, 1:32,768; IgM phase 1, 1:256; and IgM phase 2, 1:128.

## Conclusions

We report a case-patient with *C. burnetii* endocarditis and meningitis confirmed by 16S rRNA sequencing of resected valvular lesions and a *C. burnetii*–specific PCR of cerebrospinal fluid. Although not confirmed, we suspect that his episode of bronchitis 3 months earlier, which occurred a few weeks after visiting a petting zoo, might have represented atypical pneumonia caused by *C. burnetii*.

The primary reservoirs for *C. burnetii* are goats, sheep, and cattle (*1*). The most common mechanism of infection in humans is inhalation of aerosolized bacteria resistant to environmental stress; however, consumption of poultry or raw or undercooked eggs are other possible routes (*1*). The organism has 2 distinct antigenic phases (phase 1 and phase 2); the immune response to acute infections is predominantly against phase 2, and the response to chronic infection is predominantly against phase 1, although there can be major increases in titers against both phases (*2*).

Acute *C. burnetii* infection is most commonly asymptomatic but can cause symptoms ranging from an influenza-like illness to pneumonia or hepatitis with varying degrees of severity (*3*,*4*). Progression from acute to chronic Q fever occurs in ≈1%–5% of case-patients (*5*,*6*). Chronic Q fever most commonly manifests as endocarditis (60%–70% of cases), which most often occurs in the setting of a valvular lesion (*4*,*7*). This case did not have a known valvular lesion. We believe that the bicuspid valve was likely colonized during the acute phase and resulted in a transition to chronic Q fever endocarditis.

Meningitis is a rare manifestation of chronic infection with *C. burnetii* (*4*). One case series identified a male predominance among persons given a diagnosis of Q fever meningitis and reported a lymphocytic predominance in cerebrospinal leukocyte counts (*8*), as observed for this case-patient. A previous report of concomitant Q fever endocarditis and meningitis attributed central nervous system involvement to embolic phenomenon from a valvular vegetation (*9*). The cause of Q fever meningitis was unclear for the case-patient we report.

Data are limited regarding the prevalence of chronic Q fever in the United States. The Centers for Disease Control and Prevention reported 160 cases of *C. burnetii* infection in the United States during 2014, of which 39 were diagnosed as chronic Q fever (*10*).

Diagnostics for *C. burnetii* are limited because this bacterium is difficult to culture (*11*). In patients with risk factors (e.g., the case-patient we report) and culture-negative endocarditis, *C. burnetii* endocarditis should be considered. Complement fixation is a standard serologic test for *C. burnetii* at the University of California, Los Angeles. Immunofluorescence assays, which are standard tests at many institutions (*12*), were not available. Diagnostic methods include immunohistochemical analysis of resected heart valves, serologic studies, and qualitative PCRs on freshly resected heart tissue (*13*).

The 16S rRNA gene has regions that are highly conserved across bacteria but with sufficient sequence differences to enable genus, if not species, differentiation (*14*). Thus, use of 16S rRNA sequencing offers a useful diagnostic approach. However, because those regions are highly conserved, 16S rRNA sequencing is not the most sensitive or specific diagnostic approach, but might be most useful when the bacterial cause is unknown.

In summary, *C. burnetii* endocarditis and meningitis should be considered in cases of culture-negative disease for patients with appropriate risk factors. Use of 16S rRNA sequencing might aid in diagnosis of infection.
